# Valorization of pineapple processing residues through acetification to produce specialty vinegars enriched with red-Jambo extract of *Syzygium malaccense* leaf

**DOI:** 10.1038/s41598-022-23968-2

**Published:** 2022-11-12

**Authors:** Fernanda Aparecida Brocco Bertan, Eduardo da Silva Pereira Ronning, Marcelo Luis Kuhn Marchioro, Tatiane Luiza Cadorin Oldoni, Robert F. H. Dekker, Mário Antônio Alves da Cunha

**Affiliations:** 1 Programa de Pós-Graduação Em Biotecnologia, Universidade Tecnológica Federal Do Paraná, Dois Vizinhos, Paraná Brazil; 2 Departamento de Química, Universidade Tecnológica Federal Do Paraná, Pato Branco, Paraná Brazil; 3 Programa de Pós-Graduação Em Tecnologia de Processos Químicos E Bioquímicos, Universidade Tecnológica Federal Do Paraná, Pato Branco, Paraná Brazil; 4 Beta-Glucan Produtos Farmoquímicos-EIRELI, Lote 24A - Bloco Zirconia, Universidade Tecnológica Federal Do Paraná, Avenida João Miguel Caram, 731, Londrina, Paraná CEP: 86036-700 Brazil

**Keywords:** Biotechnology, Microbiology

## Abstract

The present study proposes the production of vinegars from pineapple processing residues as an eco-friendly strategy for adding value and economic strengthening of the production chain. Pineapple pulp and peel wines were produced and acetificated to vinegar by wild strains of acetic bacteria using Orlean’s method (traditional system) followed by enrichment with leaf extract of Red-Jambo, *Syzygium malaccense.* Appreciable phenolic contents and antioxidant potential were found in pulp and peel vinegars with the added leaf extract. Catechin, epicatechin and caffeic, *p*-coumaric, ferulic, and gallic acids were the main phenolic compounds found in peel vinegar. The enrichment of the vinegar with the extract promoted an increase in the content of polyphenols (443.6–337.3 mg GAE/L) and antioxidant activity. Peel wines presented higher luminosity (L^*^) and higher saturation index (C^*^), and their color tended more toward yellow than pulp wines. Acetification reduced the saturation index (C^*^) and led to the intensification of the hue angle in the peels vinegar. Each type of pineapple vinegar produced showed biocidal activity against different bacteria and yeast, and the addition of leaf extract potentiated the antimicrobial activity of peel vinegar, especially against *Staphalococcus aureus*. The vinegars developed could find an attractive market niche in the food sector.

## Introduction

Vinegar is an ancient and versatile product whose production has been known for at least 5000 years. Its origin is probably associated with the production of the first wines, which gave rise to the product by the natural oxidation of ethanol to acetic acid^1^. Vinegar is a condiment used not only as a seasoning but in preserving the color, odor, and quality of processed foods, such as canned vegetables, ketchup, mustard, and sauces among others. In addition to the food sector, vinegar is also used to clean environments, neutralize odors, eliminate mites, and remove encrustations from domestic objects, among other purposes^2^.

**Figure 1:**
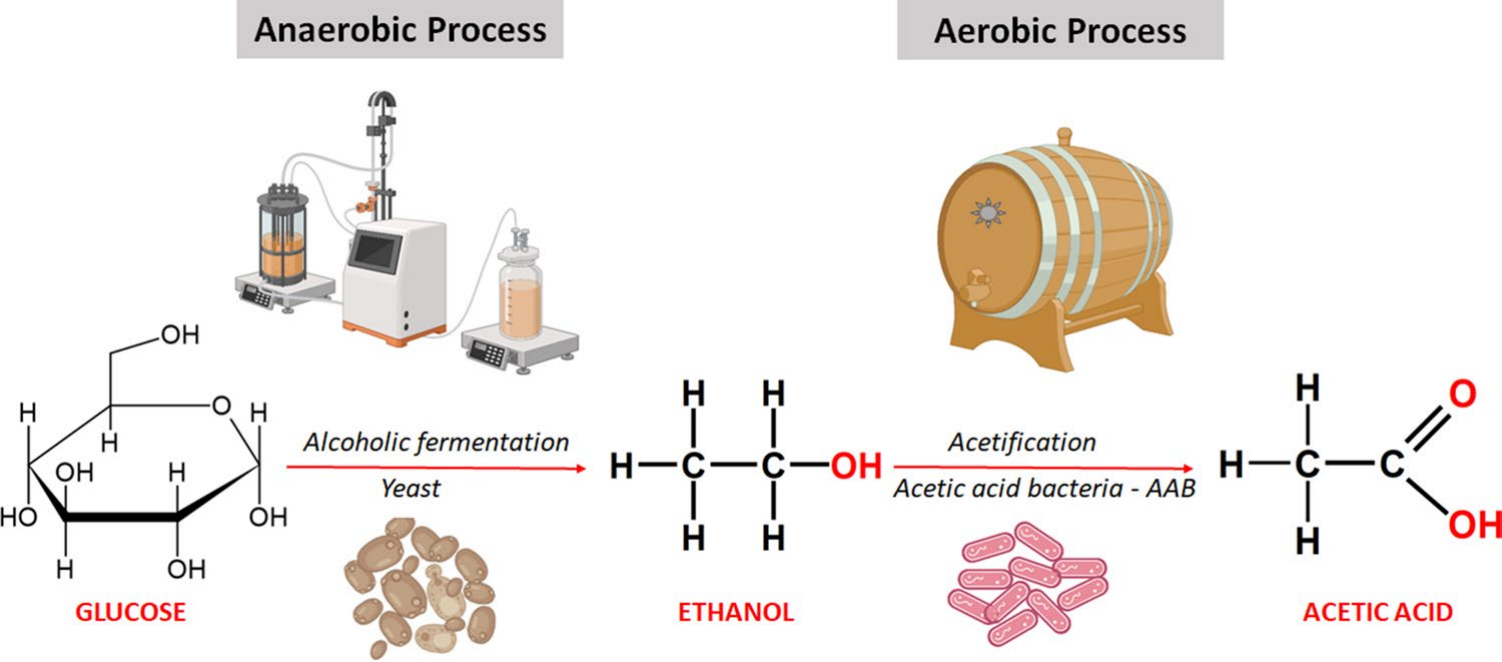
Steps involved in the bioprocess of vinegar production in submerged systems (Figure Created with BioRender.com).

The production of vinegar takes place through two traditional biotechnological processes. The first corresponds to the alcoholic fermentation carried out by yeasts of the genus *Saccharomyces*, which converts the fermentable sugars of the must into ethanol. The second process involves the oxidation of ethanol to acetic acid by acetic acid bactéria. Apparently, species of the genera *Acetobacter* and *Komagataeibacter* (many of them relocated from the genus *Gluconacetobacter*) are dominant in the acetification process, due to their high tolerance to acetic acid and preference for ethanol^3,4^. Such genera are characterized by the ability to convert ethanol into acetic acid by oxidation, as shown in the scheme described in Fig. 1.

Vinegar can be obtained from different raw materials, such as fruits, cereals, honey, and ethanol, and the product can also be produced from a mixture of these. Solid state fermentation is traditionally used in Asia for commercial vinegar production. Cereals such as sorghum and rice are used as raw materials in obtaining traditional vinegar in China, such as *Shanxi*-aged vinegar, *Zhenjiang*-aromatic vinegar and aged-vinegar from *Tianjin Duliu*^1,5^. In the fermentation of Chinese cereal vinegars, substrate degradation and fermentation are allowed to occur simultaneously, with the microbiota changing continuously throughout the process, which comprises saccharification, alcoholic fermentation and acetification^1,6^. Submerged fermentation is most widespread in Europe^7,8^ and other western countries, which allows vinegar production on a larger scale.

The most consumed vinegar in Brazil is that obtained from ethanol, wine, and apple cider by submerged fermentation. However, fruit vinegar recently has attracted the attention of consumers due to the functional benefits arising from its antioxidant, antimicrobial, antiallergic, and hepatoprotective activity. Fruit vinegars possess several nutrients, including amino acids, sugars, vitamins, and minerals, which provide energy, regulate cellular metabolism, and immunomodulation, among others. In addition, fruit vinegar products contain bioactive components such as organic recognized antioxidant activity; they also regulate lipid metabolism and blood pressure, control glucose levels, and offer liver protection, in addition to having anti-fatigue and antitumor properties^1^. Acids, polyphenols, melanoidins, and tetramethylpyrazine with.

Another relevant aspect of fruit vinegar production is the possibility of using surplus fruit as well as fruit residues, such as peels and stalks^9^. Among fruits are pineapples (*Ananas comosus* [L.] Merr.) that belong to the Bromeliaceae family, and are a good example considering their worldwide production and consumption. The consumption of pineapples in the minimally-processed form, either as juice or sliced and canned has grown in recent years. Consequently, there is an increase in the generation of processing residues that are basically constituted of peels and pomace. Such residues still have a certain sugar content and are rich in bioactive compounds, and therefore, could be used to obtain *new* value-added products, including specialty vinegars^10^.

Pineapple peels make up 37% of the fruit, and only a tiny amount of this biomass residue is used commercially, e.g., as fertilizer, or as an animal feed. Pineapple peel waste has also been used as a raw material in extracting pectin, as a fermentation substrate for the production of solvents (ethanol, butanol), and biogases (hydrogen, methane), as well as sources of bromelain (protease enzymes), phenolic flavor compounds, antioxidants, and organic acids^11^. Peels offer a possible source for extracting bioactive compounds such as polyphenols, and antioxidants for use in *intelligent* packaging^12^. Moreover, pineapple peel extracts have recently been used as a low-cost electrolyte in the remediation process of chromium-contaminated soils^13^, and has also demonstrated anti-malarial, anti-nociceptive, and anti-inflammatory activities^14^.

A strategy still little explored by the vinegar industry is the enrichment of the content of functional compounds in vinegars, which can easily be achieved by adding plant extracts rich in bioactive compounds. In this context, the red-Jambo extract of *Syzygium malaccense* (L.) Merr. & L.M. Perry) leaves, is rich in bioactive compounds and could be used as an additive to enhance the quality of unique vinegars. Red-jambo leaves are rich in flavonoids such as catechin, mearnsitrin, myricitrin, quercitin, and the anthocyanins: cyanidin-3,5-*O*-glucoside, cyanidin-3-*O*-glycoside and peonidin-3-*O*-glucoside^15^.

*S. malaccensis* (L.) Merr. & Perry belongs to the Myrtaceae family of Asian origin (India and Malaysia), but is also found in Australia, the Caribbean, and Brazil. The species grows rapidly reaching 12- to 15- meters in height, possessing a pyramidal or cylindrical crown, being used ornamentally for its beauty and being able to bloom 2 to 3 times a year in Brazil and the Caribbean varieties^16^.

There are almost no scientific reports and information about enriching the quality of fruit vinegars with extracts or tinctures from plants and herbs. Therefore, the objective of the present study was the elaboration and characterization of vinegars made from pineapple pulp and peels to which was added an extractive of red-Jambo leaves.

The focus of this work is on using the solid waste from processing pineapples to obtain value-added products (gourmet vinegars) with perspectives of sustainability, and strengthening of the circular bioeconomy. A new alternative value-added product is proposed for the management and direction of solid processing waste generated at both small and large pineapple processing companies, and companies producing fruit juices or those selling the fruit in the minimally-processed form. Adding value to this processing waste stream means strengthening the three basic pillars of sustainable development, which include attention to the economy, society and the environment.

We herein report on the vinegars produced from pineapple pulp and peel with added leaf-extract of *S. malaccense* (L.) which are rich in phenolic acids and flavonoids and possess antimicrobial activity. The vinegars obtained could potentially contribute to nutritional and health benefits for the consumer.

## Material and methods

### Microorganisms, materials, and red-Jambo leaf extract

*Saccharomyces cerevisiae f. r. bayanus* (Fermol Perlage) used in this study was provided by the company AEB Biochemistry Latin American AS (Brazil). The wild culture of acetic acid bacteria used in the acetification of wines was isolated from colonial red grape (*Vitis labrusca*) vinegar produced in the southwestern region of Paraná, Brazil^17^. Analytical reagents and culture media were purchased from Merck S/A (Brazil) with adequate purity grades. Chromatographic standards (HPLC grade, with purity ≥ 99%) of phenolic acids and flavonoids were purchased from Sigma-Aldrich (St. Louis, MO, USA).

Pineapple peels were obtained in the laboratory from healthy and ripe fruits (Smooth Cayenne variety) acquired in the municipality of Pato Branco (Paraná, Brazil). The fruits were sanitized in sodium hypochlorite solution (100 ppm, 15 min), rinsed in running water, and the peels were then separated from the pulp using stainless steel knives.

Red-Jambo (*Syzygium malaccense*) leaves were dehydrated at 35 °C, and the extract was obtained by subjecting the leaves to hydroalcoholic extraction (40% ethanol: 60% water) at 80 °C for 45 min as previously optimized by Savi et al.^18^. The extract was concentrated in a rotary evaporator to eliminate ethanol and lyophilized it for later use.

### Inoculum preparation for alcoholic and acetic fermentation

The commercial yeast *Saccharomyces cerev*isiae r.f. bayanus was rehydrated and cultivated in malt-extract broth (20 g/L malt extract, 1 g/L peptone, and 20 g/L glucose) in 250-mL Erlenmeyer flasks (100 mL of culture medium) in an orbital incubator (shaker) at 28 °C, 150 rpm for 24 h. The cultured cells were recovered by centrifugation (1500 × *g*, 15 min), washed with sterile isotonic saline solution, transferred to flasks containing 90 mL of freshly prepared malt-extract broth, and cultured for 12 h (28 °C, 150 rpm). The pre-inoculum yeast cells were recovered by centrifugation (1500 × *g*, 15 min), washed, and resuspended in 300 mL of pineapple juice to obtain a concentration of 1 × 10^6^ cells/mL, which was used as the inoculum in the alcoholic fermentation step^17^.

Acetic acid bacteria were isolated from unpasteurized vinegar (strong vinegar) acquired from a small rural property in Pato Branco, Paraná. A volume of 10 mL of strong vinegar was transferred to 100 mL Erlenmeyer flasks containing 90 mL of G-Y medium (100 g/L glucose, 10 g/L yeast extract) supplemented with 100 mg/L of natamycin to inhibit the growth of yeasts and fungi. The bacterial culture was kept in an orbital incubator (shaker) for 24 h under agitation (150 rpm), and at 28 °C. The cultured cells were next separated from the medium by centrifugation (1500 × g, 15 min), washed with sterile saline solution (0.9 g NaCl/100 mL), resuspended in 20 mL of distilled water, and then used as the inoculum in the acetification of pineapple pulp and peel wines^17^*.*

### Obtaining the must and fermentation

Pineapple fruit peels were cut into strips and processed in a blender using a ratio of peels to drinking water of 1:3 (w/v). The fruit pulp was cut into cubes and crushed in a blender at maximum speed until complete liquefaction. The pH of the broths obtained from the peel or pulp was adjusted to 4.0 with 1 mol/L NaOH solution and submitted to enzymatic hydrolysis using the enzyme complex Pectinex Ultra SP-L® (3800 PGNU/mL; Novozymes, Bagsvaerd, Denmark) in the proportion of 15 mL per 100 g of peel or pulp, and incubated at 30 °C for 70 min under agitation (150 rpm).

After hydrolysis, both broths were heated at 90 °C for 5 min to terminate enzyme activity, and the hydrolyzate cooled in an ice-bath. The total soluble solids content of the broths was corrected (chaptalization) with sucrose to 18°Brix. The chaptalized broths were supplemented with Enovit (30 g/hL, a commercial yeast growth activator) and pasteurized at 65 °C for 30 min to obtain chaptalized musts used in alcoholic fermentation.

The alcoholic fermentations were carried out in a fermenting bucket with a coupled S-shaped airlock under static conditions at 28 °C. A volume of 2.7 L of must and 300 mL of inoculum (2 × 10^6^ yeast cells/mL) was used in each fermentation run, and samples removed at 24-h intervals for analyzes (see below).

The wines produced were separated from the lees, peel, and pulp by centrifugation (1500 × *g*, 20 min), supplemented with the mineral complex Acetozyn® (1.5 g/L. Heinrich Frings GmbH & AMP; Co, USA) and transferred into a 2.5-L Grapia barrel for the acetic fermentation step. The total working volume was 1500 mL, and the inoculum volume was 150 mL, as previously described by Fonseca et al.^17^. The barrels used for acetification were kept at 25 °C during the acetic oxidation step. The process was accompanied by determining the pH, total acidity expressed as acetic acid (%), and acetic acid content (g/L).

The vinegars produced were subjected to centrifugation under refrigeration (1500 × *g*, 30 min at 4 °C) for clarification and then enriched with the lyophilized red-Jambo leaf extract of *Syzygium malaccense* (500 mg of extract/L). The final product was placed in 250 mL glass bottles and then subjected to slow pasteurization in a water bath at 62 °C for 30 min^19^.

### Analytical determinations

The lyophilized red-Jambo leaf extract was characterized in terms of total phenolic content by the Folin–Ciocalteau spectrophotometric method^20^, and antioxidant potential by the radical scavenging methods: ABTS^21^, DPPH^22^ and ferric reducing antioxidant power (FRAP)^23^.

The alcoholic fermentation runs were sampled every 24 h, and determinations were conducted for total soluble solids (manual refractometer), pH (pH meter), titratable acidity (235/IV method)^24^ , and total reducing sugar content by the method DNS^19^, ethanol concentration (HPLC), and yeast cell counts were performed on plating the fermentation broth on Sabouraud-agar plates. The wines obtained were also characterized in terms of titratable acidity (235/IV method)^24^, and total phenolic content (Folin–Ciocalteau method), bioactive compounds (HPLC), and antioxidant activity (ABTS, DPPH and FRAP methods).

The vinegar samples were analyzed for pH (pH meter), total acidity (504/IV method), mineral residues (incineration at 550 °C), reduced dry extract (509/IV method), density (specific weight at 20 °C)^24^, total sulfur dioxide content by the Ripper method^25^ levels of acetic acid and ethanol (HPLC), total reducing sugars^19^ total phenolics^20^, bioactive compounds (HPLC), antioxidant activity (ABTS, DPPH and FRAP methods), color by the CIELab system (L * a * b* color space).

Antimicrobial activity was evaluated by the disc-diffusion method and broth microdilution to determine the minimal inhibitory concentration (MIC) against microbial strains of clinical importance^18^.

Phenolic acids and flavonoids were analyzed by HPLC–DAD using a 920 LC chromatographic system (Varian Inc., Walnut Creek, CA, USA), equipped with a C18 column. The column was kept at 30 °C during the analysis, and the injection volume of the samples (wine, vinegar, or properly diluted red-Jambo extract) was 10 μL. The mobile phase consisted of a gradient mixture of 2% (v/v) aqueous acetic acid solution (Solvent A), and 40% (v/v) acetonitrile acidified with 2% aqueous acetic acid solution (Solvent B) with a flow rate of 1 mL/min. The gradient commenced with 5% solvent B adjusted to 20% and run for 2 min, 25% B for 15 min; 85% B for 25 min and then held for 5 min; 20% B at 33 min; 5% B for 16 min with an 8 min conditioning step. Peak areas were determined at 280 nm for gallic acid, vanillic acid, and flavonols: catechin and epicatechin; 300 nm for coumaric acid and salicylic acid; 320 nm for caffeic and ferulic acids and 360 nm for flavonoids: rutin and quercetin.

The contents of ethanol, acetic acid, and other organic acids present in the wine and vinegar samples were determined by HPLC (920 LC chromatographic system equipped with a refractive index detector) and a column HPX-87-H (Bio-Rad, Hercules, CA) maintained at 45 °C, using sulfuric acid solution (0.005 mol/L) as the eluent, at a flow rate of 0.4 mL/min and injection volume of 20 µL per sample. The samples appropriately diluted were first passed through a 45 µm CHOMAFIX filter, and in SEP PACK C18 cartridges (Waters Corporation). Compounds were identified by comparing retention times with authentic standard samples, and quantified by integrating the areas of the respective peaks.

### Color analysis

For color evaluation, the samples were filtered through 0.45 µm membrane filters, and the color was analyzed in a CR-410 digital colorimeter (Konica Minolta, Japan) using the CIELAB color space. The parameters: luminosity (L^*^), the chromaticity coordinates a^*^ and b^*^, and the cylindrical coordinates: hº (hue angle, tonality), and C^*^ (chroma) were measured. After filtering, the samples were placed in the instrument's cuvette, and the measurements were taken in sequence. The color difference between the samples was determined from the Equation bellow.

1 $$\Delta E * = \, (\Delta L^{{ * }{2}} + \Delta a^{{ * }{2}} + \, \Delta b^{{ * }{2}} )^{{{1}/{2}}}$$

where ΔE corresponds to the total color difference between the samples; ΔL^*^ is the difference in brightness, Δa^*^ is the difference in red and green (+, redder; −,  greener), and Δb^*^ = the difference in yellow and blue (+, more yellow; −, bluer).

### Statistical analysis

The results were presented as mean ± standard deviation, and the Kolmogorov–Smirnov test confirmed the normal distribution of variables. The values ​​of the characterization parameters studied in the wines of pulp and peel were compared by the Student's t-test (*p* < 0.05) using the GraphPad Prism® 8 program (GraphPad Software, Inc.). The characterization parameters of the vinegars were submitted for analysis of variance, and the means were compared by the Tukey test at a significance level of 5% (*p* < 0.05). OriginPro 8.5 software (OriginLab Corporation, Northampton, MA, USA) was used to create the profile graphs of the fermentations.

### Consent to participate

All authors have their consent to participate.

## Results and discussion

### Total phenolics and the antioxidant potential of Syzygium malaccense leaf extract

Table 1 presents the total phenolic content and antioxidant potential found in the red-Jambo leaf extract that was added to the vinegars produced in the present work.

**Table 1:** Total phenolics and antioxidant activity in red-Jambo leaf extract.

Bioactivity parameters	Values
Total phenolics (mg gallic acid equivalents/10 g)	385.40 ± 0.03
**Antioxidant activity**	
ABTS (µmol trolox equivalent/10 g)	41.83 ± 0.01
DPPH (µmol trolox equivalent/10 g)	11.82 ± 0.02
FRAP (µmol FeSO_4_/10 g)	54.15 ± 0.05

Extracts of red-Jambo leaves are still little used. Recent evidence reported that its leaves are rich in phenolic compounds and flavonoids, in addition to having antioxidant activity^18^. A high content of total phenolics (385.40 mg Gallic Acid Equivalents—GAE/10 g) was found in the lyophilized red-Jambo leaf extract. However, values ​​somewhat higher (537.70 mg GAE/10 g) were reported by Batista et al.^26^ in the red-Jambo leaf extract obtained using methanol. Different parameters including genetic aspects of the plant, the time and period/season of harvesting, and agronomic conditions related to the crops of the plant, among others, can contribute to obtaining extracts with varying contents of total phenolics.

The antioxidant activity of polyphenols is attributed to their Redox properties, which allows them to act as reducing agents, hydrogen donors, singlet oxygen scavengers, and in metal-chelation^27^. In this context, appreciable scavenging potential of DPPH (11.82 µmol Trolox equivalent—TE/10 g) and ABTS (41.83 µmol TE/10 g) radicals were found in the present work. Similarly, the leaf extract showed a ferric ion reduction capacity of 54.15 µmol FeSO_4_/10 g. The values ​​found in the present study are much higher than those reported previously by Savi et al.^18^ in a similar extract (ABTS: 0.853 µmol TE/kg, DPPH: 0.666 µmol TE/kg, FRAP: 1.267 µmol TE/kg).

### Fermentation profile of pineapple must vinification

**Figure 2:**
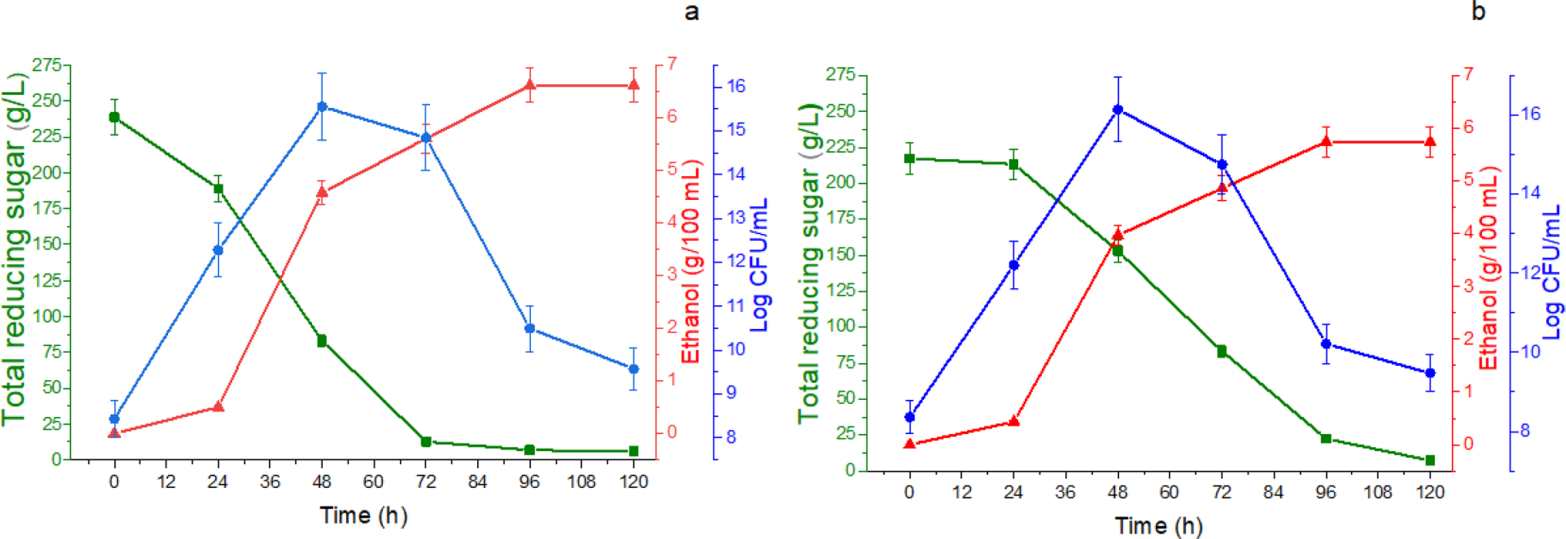
Alcoholic fermentation of musts from (**a**) pineapple pulp and (**b**) pineapple peel showing the kinetic profile of reducing sugars (green filled square box), ethanol production (red filled uptriangle), and cell growth (blue filled circle) in the winemaking process.

The profiles of alcoholic fermentations of musts based on pineapple pulp and peel are shown in Fig. 2a,b. The fermentation time was set at 120 h when the release of CO_2_ ended, and consumption of at least 95% of the sugar content of the musts had occurred. The inoculum used in alcoholic fermentations exhibited high cellular activity concerning growth, substrate consumption, and fermentative activity.

The hydration and cultivation of commercial yeast in malt-extract broth and subsequent suspension in pineapple juice resulted in obtaining a metabolically-active inoculum, which was verified by the absence of the lag phase of yeast growth. In this sense, a linear increase in the number of yeast cells was observed during the first 48 h of cultivation, when the maximum cell density (≅ Log 16 CFU/mL) was obtained both in the musts of pineapple pulp (Fig. 2a), and pineapple peel (Fig. 2b).

Cell growth was accompanied by effective substrate consumption and ethanol production, and especially in the pineapple-pulp cultivated cultures. In 72 h of the pulp fermentation, 94.73% of the substrate was consumed, and produced an ethanol concentration of 56.04 g/L. On the other hand, lower values ​​of substrate assimilation (61.60%) and ethanol production (48.61 g/L) were observed in pineapple peel-based musts (72 h). At the end of the alcoholic fermentations, similar values ​​of consumption of total reducing sugars were verified in musts obtained from the pulp (97.60%) and peel (96.70%). The yeast used in the present work also showed similar substrate assimilation values (Y_C_: 96.50%) in must formulated with blackberry and honey as previously described by Fonseca et al.^17^.

Regarding ethanol accumulation in the fermented broth, higher amounts were observed in musts formulated with the pulp (66.20 g/L) than with the peel (57.40 g/L). The substrate assimilation profile by the yeast observed in 72 h of fermentation suggests that the hydrolyzed pineapple peels present in the must hindered the assimilation of sugars. Such behavior may be related to the complexity of the chemical composition of the pineapple peels. In fact, in addition to the fermentable sugars (glucose, fructose, sucrose) present in the peels, were high contents of structural polysaccharides (cellulose, hemicellulose, pectin) and lignin^28^, as well as, phenolic compounds, alkaloids, flavonoids, tannins and saponins, and other secondary compounds, which reportedly can have antimicrobial potential^29,30^.

The fermentative parameters of yeast cultivation in medium based on pineapple pulp and peel are described in Table 2. Determining such parameters is essential to evaluate the efficiency and yield of alcoholic fermentation, allowing a better understanding and comparison of the process.

**Table 2:** Fermentation parameters of pineapple pulp and peel must vinification.

Fermentation parameters	Wines–alcoholic fermentation	
	Pulp	Peel
Ethanol production (P)	66.20 g/L ± 2.11	57.40 g/L ± 1.88
Ethanol yield (Y_P/S_)	0.28 g/g ± 0.01	0.27 g/g ± 0.01
Volumetric productivity in ethanol (Q_P_)	0.55 g/L.h ± 0.03	0.48 g/L.h ± 0.02
Overall substrate consumption rate (Q_S_)	1.94 g/L.h ± 0.07	1.75 g/L.h ± 0.05
Efficiency of alcoholic fermentation (η)	54.80% ± 0.00	52.80% ± 0.00
Overall percentage of substrate consumption (Y_C_)	97.50% ± 1.20	96.70% ± 1.10
Maximum specific growth rate (µmáx)	0.37 h^−1^	0.34 h^−1^

As observed with the assimilation of fermentable sugars, the ethanol content accumulated at the end of the fermentation run was 15.30% higher in the broth fermented with pineapple pulp compared to that of the peel. Similarly, Alvarenga et al.^31^ reported that the addition of pineapple peels in musts formulated with the pulp contributed to a reduction in ethanol production. Roda et al.^9^, and Chalchisa and Dereje^32^ also reported lower ethanol production values in wines produced from musts formulated with pineapple peels (47.34 g/L and 47.02 g/L, respectively). It is important to note that although greater ethanol production was obtained with the pulp wines, the ethanol yields (substrate to product conversion factor) were similar in both the pulp (Y_P/S_: 0.28 g/g) and peel (Y_P/S_: 0.27 g/g) wines. The alcoholic fermentation efficiency (η) parameter shows a conversion efficiency of 54.80% (1 g of sugar generated 0.28 g of ethanol) of the assimilated sugars into ethanol in the vinification of pineapple pulp must. Similarly, a conversion efficiency of 52.80% (1 g of sugar generated 0.27 g of ethanol) of the assimilated sugars to ethanol was found in the fermentation of must based on pineapple peels. The overall percentage of substrate consumption (Y_C_) was also similar under both fermentation conditions (97.50% and 96.70%). Likewise, little difference was found concerning the process efficiency parameter values (η: 54.8% and 52.8%). Regarding the overall substrate consumption rate (Q_S_), fermentation with pulp showed values 10.9% higher than those found in fermentation with peel. Corroborating the substrate assimilation profile in the exponential growth phase, the maximum specific rate of yeast growth was slightly higher in fermentation with the pulp must (µ_max_: 0.37 h^−1^), suggesting that during this phase, a higher percentage of the substrate was directed to yeast cell growth in fermentation of the pulp compared to the peel (µ_max_: 0.34 h^−1^).

The fermentation results show that pineapple peels have potential as a raw material for formulating musts intended for alcoholic fermentation. Although the musts formulated with pure pulp stood out in fermentation, the peels showed good fermentative capacity.

Table 3 describes the physical–chemical and bioactivity parameters of wines made with pineapple pulp and peel.

**Table 3:** Physicochemical and bioactive parameters of pineapple pulp and peel wines.

Parameter analyzed	Wines–alcoholic fermentation	
	Pulp	Peel
pH	3.40^a^ ± 0.01	3.69^b^ ± 0.01
Titratable acidity (g/100 mL)	7.60^a^ ± 0.01	4.60^b^ ± 0.06
Total reducing sugars (g/L)	5.86^a^ ± 0.01	7.24^b^ ± 0.00
Ethanol % (v/v)	8.39^a^ ± 0.02	7.28^b^ ± 0.01
Density (kg/m^3^)	987.0^a^	969.0^b^
Free sulfur dioxide – SO_2_ (mg/mL)	nd	nd
Total sulfur dioxide – SO_2_ (mg/mL)	nd	nd
**Phenolic compounds**		
Total phenolics (mg GAE/L)	188.97^a^ ± 0.00	110.53^b^ ± 0.02
Catechin (mg/L)	31.63	27.88
Caffeic acid (mg/L)	< DL	1.73
*p*-Coumaric acid (mg/L)	0.35	4.05
Ferulic acid (mg/L)	< DL	1.48
**Organic acids**		
Ascorbic acid (g/L)	2.70	1.90
Citric acid (g/L)	6.20	1.00
Malic acid (g/L)	1.90	0.70
Oxalic acid (g/L)	7.80	4.91
Succinic acid (g/L)	2.80	1.20
**Antioxidant activity**		
ABTS (µmol TE/100 mL)	274.0^a^ ± 0.0	211.0^b^ ± 0.0
DPPH (µmol TE/100 mL)	129.0^a^ ± 0.0	139.0^b^ ± 0.0
FRAP (µmol FeSO_4_/100 mL)	562.60^a^ ± 0.08	258.10^b^ ± 0.09
**Color**		
L*	42.73^a^ ± 0.10	52.75^b^ ± 0.01
a*	− 0.78^a^ ± 0.15	− 0.16 ^b^ ± 0.01
b*	2.61^a^ ± 0.24	13.20^b^ ± 0.01
H^º^	110.90^a^ ± 3.53	90.70^b^ ± 0.03
C*	2.42^a^ ± 0.22	13.20^b^ ± 0.01
ΔE	14.59	

In Table 3 (GAE: gallic acid equivalent, TE: trolox equivalents (Trolox-Equivalent Antioxidant Capacity), ABTS: 2,2'-azino-bis(3-ethylbenzothiazoline-6-sulfonic acid), DPPH: 2,2-Diphenyl-1-picrylhydrazyl, FRAP: ferric reducing antioxidant power, nd: not detected, < DL: values below detection limit, L*: luminosity, a*[(−) green to (+) red] and b* [(−) blue to + yellow] chromaticity coordinates, ho, Hue angle and C*, Chromaticity, ΔE: Total color difference. Different letters on the same line differ statistically at the 95% confidence interval (*p* < 0.05).

Pulp and peel wines had pH values ​​of 3.94 (pulp) and 3.69 (peel) and titratable acidity expressed in acetic acid of 0.76 g/100 mL (pulp) and 0 0.46 g/100 mL (peel). Final pH values ​​between 3.5 and 4.0 were reported by Cunha et al.^33^ in blackberry wines and Fonseca et al.^17^ in mixed blackberry and honey wines produced in a vinification process where the same yeast used in the present study was also used. The acidity of a wine is basically due to the presence of organic acids from the fruit itself, such as malic acid, tartaric acid, citric acid, and the production of acids during fermentation, such as acetic acid^34^. Both pineapple wines obtained showed prominent acidity, especially the pulp wine, possibly because it contained higher amounts of organic acids derived from the fruit itself. Qi et al.^34^ obtained pineapple wines with lower acidity (0.229 g/100 mL) than was found in this work. This can be attributed both to the characteristics of the fruit and the metabolic properties of the yeast used in the winemaking process. Low pH values ​​during alcoholic fermentation can prevent the growth of undesirable microbiota; an advantage that enhances the quality of the final products.

The alcohol contents present in the pulp and peel wines were 8.39% (v/v) and 7.28% (v/v), respectively. Such values ​​are higher than those reported by Alvarenga et al.^31^ in wine from the musts formulated with pineapple pulp (6.8% v/v) and pulp plus peel (100 g/kg) (5.9%, v/v). Lower ethanol values ​​were also reported by Roda et al.^9^ in wines of pineapple peel (6.0%, v/v). It is important to point out that the ethanol content present in wines destined for acetification is a parameter of importance that must be analyzed and adjusted where necessary.

Very high ethanol content in wines can lead to vinegars with a high acetic acid content, resulting in a very acidic product that does not meet the legislation standard. Associated with excess acidity, vinegars with ethanol contents outside the legislation standard can also be produced. Chalchisa and Dereje^32^ reported the concentration of ethanol in wine should be less than 7.5% to obtain a good quality vinegar. However, it is important to consider the acetification process used, the acetic acid bacteria used in the ethanol-to-acetic acid bioconversion, as well as ethanol losses incurred by evaporation during the acetification process.

Pineapple pulp and peel wines showed density values ​​of 987 and 969 kg/m^3^, respectively, that agreed with those reported by Akanni Ahoussi et al.^35^ in pineapple wine (995.0 kg/m^3^). Similar values ​​(998.2 kg/m^3^) were also found by Queiroz et al.^36^ in alcoholic-fermented pineapple juice. The density of the wine varied according to the amount of sugars and ethanol present in the product.

Brazilian legislation^37^ establishes a maximum limit to the addition of 300 mg/L of sodium metabisulfite in musts that are destined for commercial alcoholic fermentation. No sulfurous anhydride (SO_2_) residues were detected in the pineapple wines produced in the present study, which was to be expected as the musts were not sanitized with metabisulfite prior to the fermentation stage. Slow pasteurization was the method that we chose to sanitize the musts before alcoholic fermentation.

As shown in Table 3, appreciable total phenolic content was found in both the pulp wine (188.97 mg GAE/L) and peel wine (110.53 mg GAE/L). Different contents of total phenolics have been reported in the scientific literature. Pino and Queris^38^, when evaluating the content of total phenolic compounds in pineapple wines, found lower values ​​(108.0 mg GAE/L) than those found in the present work. On the other hand, Zhang et al.^39^ found higher values in pineapple peel wine (675.43 mg GAE/L). The different phenolic contents may be associated with the origin of the fruit, the degree of maturation, and the vinification process used^39^.

**Figure 3:**
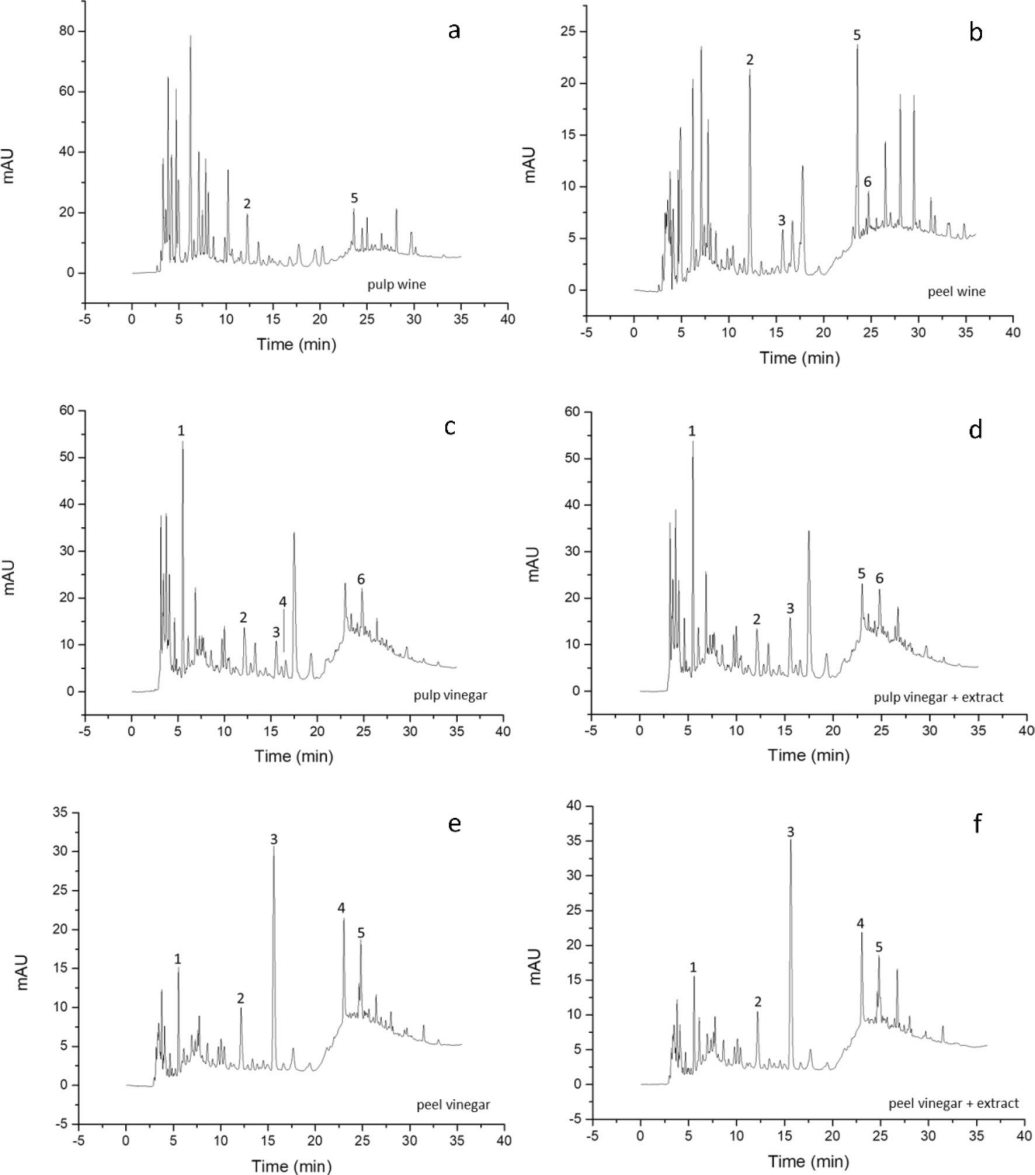
HPLC–DAD chromatograms for samples of (**a**) pulp wine, (**b**) peel wine, (**c**) pulp vinegar, (**d**) pulp vinegar + extract, (**e**) peel vinegar, (**f**) peel vinegar + extract. Gallic acid (1), catechin (2), caffeic acid (3), epicatechin (4), coumaric acid (5), ferulic acid (6).

The use of high-performance liquid chromatography with diode array detection allowed the identification (Fig. 3) and quantitation of phenolic acids: caffeic acid, coumaric acid, ferulic acid, and the flavonoid catechin in pineapple pulp and peel wines, as outlined in Table 3.

Among the phenolic compounds, the flavonoid catechin, was found in higher concentrations in both the pulp (31.63 mg/L) and peel (27.88 mg/L) wines. *p*-Coumaric acid was also found in relatively considerable amounts (0.35 mg/L and 4.05 mg/L) in both wines. On the other hand, caffeic acid (1.73 mg/L) and ferulic acid (1.48 mg/L) were identified only in the peel wine samples. Catechin (107.39 µmol/L) and ferulic acid (139.70 µmol/L) were the main compounds found in pineapple peel extracts by Li et al.^40^. Ferulic acid has been reported in pineapple wine by Roda et al.^9^ at concentrations lower (0.138 µg/L) than those obtained in the present work (1.48 mg/L), and no detectable levels were found in the pulp wine.

The pulp and peel wines, in addition to being rich in total phenolic content, showed high antioxidant potential estimated by the DPPH, ABTS, and FRAP techniques. The pulp wine had an ABTS scavenging capacity of 274.0 µmol TE/100 mL, a slightly higher value than the peel wine (211.0 µmol TE/100 mL). Similarly, appreciable DPPH scavenging potential was also observed in both pineapple wines (129.0 µmol TE/100 mL and 139 µmol TE/100 mL, respectively). Regarding the ferric ion reducing potential, pulp wine (562.6 µmol FeSO_4_/100 mL) stood out from peel wines (258.1 µmol TE/100 mL). The higher antioxidant capacity found in the pineapple pulp wines can be explained by the higher concentration of phenolic compounds and organic acids commonly found in the fruit pulp, which have antioxidant activity^41,42^. There is a lack of reports regarding the antioxidant activity of pineapple wines in the scientific literature. Higher values ​​were reported by Fonseca et al.^17^ in blueberry wine and honey for all of the antioxidant methods evaluated. Similarly, Cunha et al.^33^ also found higher values ​​in blackberry wine for the DPPH (1395.2 µmol TE/100 mL) and ABTS (2124.0 µmol TE/100 mL) methods assessing antioxidant activity.

Organic acids such as ascorbic (vitamin C), citric, malic, oxalic, and succinic acids were found in pineapple pulp and peel used in alcoholic fermentation. Citric (6.20 g/L) and oxalic (7.80 g/L) acids were the predominant organic acids in pulp wine. Oxalic acid (4.91 g/L) and ascorbic acid (1.90 g/L) predominated in pineapple peel wine. Such results show that the pulp and the peels are rich in organic acids.

It is essential to highlight that the composition and the amounts of organic acids present in pineapple fruit can vary greatly depending on the variety, as well as the stage of fruit maturation, considering, for example, that the content of organic acids in the initial stages of fruit development is directly related to the supply of substrates for respiratory processes^43^.

Regarding the color of the samples, it is important to mention that in the CIELAB space, the luminosity coordinate (L^*^) varies from black (0) to white (100); the a^*^ coordinate varies between green (-a) and red (+ a), and the b^*^ coordinate varies from blue (-b) to yellow (+ b). The hue angle (hº) starts on the + a^*^ axis (red) and is expressed in degrees: 0° corresponds to + a (red), 90° corresponds to + b (yellow), 180° corresponds to − a (green), and 270° corresponds to − b (blue). C^*^ chroma is 0 at the center of the color axis and increases with distance from it^44^. Although this method does not provide a precise definition of color, it can effectively show differences in the color of the pulp and peel wine. Instrumental color characterization showed that samples of wine from pulp and peel showed statistically significant differences (*p* < 0.05) between the color parameters L^*^, a^*^, b^*^, and chroma (C^*^). The L^*^ values ​​of both pulp and peel wines indicate a tendency towards a grayer color than white. The wine made from peel presented a higher luminosity (52.75) than the wine produced from the fruit’s pulp (42.73). Considering that luminosity is understood as the effectiveness of light in generating the sensation of brightness or clarity when perceived by the human eye, it can be mentioned that the wine sample from pineapple peel tends to be lighter than the pulp sample. In fact, visually, the peel wine sample was visually clearer than the pulp wine.

Values of the a^*^ coordinate (negative values) indicate a green direction, while the b^*^ coordinate values show a yellow trend (positive values) in both samples. The color of the peel wine, in particular, tended more towards yellow (b^*^: 13.2) than the pulp wine (b^*^: 2.61), as indicated by the values ​​of the green-yellow color coordinate. Another color aspect that differentiated the peel wine from the wine obtained from the pulp was its saturation (C^*^: 13.2), which was more noticeable than the pulp wine (C^*^: 2.24) that presented a more neutral color. Regarding tonality (hº), which is a qualitative attribute of color, there was a significant difference (*p* < 0.05) between the two samples. Corroborating the results of the parameters L^*^a^*^b^*^, C^*^, and hº, a statistically significant total difference in color was verified between the pineapple pulp and peel wine samples.

It is worth noting that there was a total difference in color between the pineapple-derived wine samples, with an ΔE of 14.58 being observed. Such behavior could be justified by the fact that both samples presented soft green-reddish tones, but with a tendency to yellow as observed by a^*^ and b^*^ coordinates, in addition to the peel wine presenting saturation values ​​5.5 times higher than the pulp.

### Acetic fermentation and pineapple pulp and peel vinegars: physicochemical and bioactive properties

**Figure 4:**
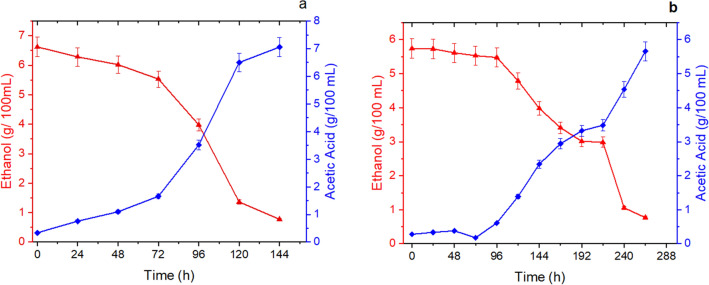
Ethanol to acetic acid bioconversion kinetic profile in the acetification of pineapple (**a**) pulp and (**b**) peel wines. Ethanol (red filled uptriangle) and acetic acid (blue filled diamond) concentrations.

The ethanol-to-acetic acid bioconversion profile of the pineapple pulp (Fig. 4a) and peel (Fig. 4b) wines reveal a good acetification efficiency of the acetic acid bacteria isolated from colonial vinegar and used as inoculum.

The acetification of the pineapple pulp wine occurred in 144 h, when a content of 7.06 g/100 mL of acetic acid, consumption of 88.30% of ethanol, 92.60% efficiency, and 0.49 g/L.h volumetric productivity in acetic acid had occurred. On the other hand, although ethanol assimilation by the acetic acid bacteria was similar in the two acetification processes (88.30% and 86.60%), a longer acetification time was observed in the fermentation of wine formulated with pineapple peels. After 264 h of acetification, the acetic acid content was 5.66 g/100 mL, corresponding to an acetic fermentation efficiency of 87.30% and volumetric productivity of 0.21 g/L h. Tanamool, Chantarangsee and Soemphol^45^ reported a maximum acetic acid content in vinegar produced from pineapple peels in processes using co-inoculation of yeasts and thermotolerant-acetic acid bacteria of 7.2% (v/v) in 16 days of cultivation.

The higher performance observed in the acetification of the pulp wine compared to the peel wine could be explained in part by the greater nutritional richness of the fruit pulp than the peel. Another aspect that can be considered is the phenolic acid composition of the peel wine. In fact, extracts containing phenolic acids commonly have antimicrobial activity, and such activity can vary considerably depending on the amounts and types of phenolic acids present^46^. Caffeic acid can interfere with the synthesis of bacterial cell wall macromolecules. Ferulic acid and catechins can modify the charge and hydrophobicity of the cell surface of gram-positive and gram-negative bacteria, leading to cell death by extravasation of the cytoplasmic material. *p*-Coumaric acid acts as an antimicrobial by disrupting the bacterial cell membrane and binding to bacterial DNA, inhibiting cellular functions^46^. Table 4 shows the physicochemical parameters of both vinegars.

**Table 4:** Physicochemical and bioactive parameters of pineapple pulp and peel vinegar.

Parameter analyzed	Vinegar			
	Pulp	Peel	Pulp + extract	Peel + extract
pH	3.64 ± 0.01	3.65 ± 0.01	3.45 ± 0.01	3.48 ± 0.01
Total acidity (g/100 mL)	5.50^b,c^ ± 0.06	4.50^b^ ± 0.04	5.58^a,c^ ± 0.06	4.73^b^ ± 0.04
Ethanol % (v/v)	0.97^a^ ± 0.10	0.61^b^ ± 0.1	0.97^a^ ± 0.10	0.97^a^ ± 0.10
Mineral residue (g/L)	4.06^a^ ± 0.01	2.40^b^ ± 0.01	3.95^a^ ± 0.01	2.52^b^ ± 0.01
Total dry extract (g/L)	30.74^a^ ± 0.01	10.93^b^ ± 0.01	31.04^a^ ± 0.01	11.47^b^ ± 0.01
Reduced dry extract (g/L)	24.88^a^ ± 0.01	6.22^b^ ± 0.01	25.13^a^ ± 0.01	6.71^b^ ± 0.01
Density (g/mL)	1.024^c^	1.026^b^	1.066^a^	1.011^b,c^
Sulfates (g/L)	nd*	nd*	nd*	nd*
**Phenolic acids and flavonoids**				
Total phenolics (mg GAE/L)	364.45^b^ ± 0.01	222.94^c^ ± 0.01	443.59^a^ ± 0.01	337.63^b^ ± 0.01
Catequina (mg/L)	23.38	12.13	27.88	12.88
Epicatequina (mg/L)	0.90	10.02	34.36	11.03
Ácido caféico (mg/L)	3.42	12.77	< DL	14.89
Ácido cumárico (mg/L)	< DL	11.60	7.61	12.02
Ácido ferúlico (mg/L)	4.85	8.46	11.56	5.88
Ácido gálico (mg/L)	18.09	3.22	19.23	3.79
**Antioxidant activity**				
ABTS (µmol TE/100 mL)	410.50^c^ ± 0.01	266.60^b^ ± 0.01	547.10^a^ ± 0.01	337.50^b,c^ ± 0.01
DPPH (µmol TE/100 mL)	216.40^a^ ± 0.01	227.80^a^ ± 0.01	249.80^a^ ± 0.01	277.50^a^ ± 0.01
FRAP (µmol Fe2 + equivalent /100 mL)	402.80^a^ ± 0.04	277.80^b^ ± 0.02	675.80^a^ ± 0.01	542.30^a^ ± 0.01
**Organic acids**				
Ascorbic acid (g/L)	1.00	0.90	1.30	0.96
Citric acid (g/L)	7.41	1.46	7.46	1.48
Malic acid (g/L)	1.80	0.20	2.00	0.27
Oxalic acid (g/L)	9.04	4.75	9.09	4.78
Succinic acid (g/L)	2.10	0.70	2.50	0.78
**Color**				
L*	45.39^a^ ± 0.01	42.29^b^ ± 0.07	45.28^a^ ± 0.03	43.12^c^ ± 0.07
a*	− 1.07^a^ ± 0.01	− 0.74^b^ ± 0.01	− 1.13^a^ ± 0.02	− 0.71^b^ ± 0.01
b*	− 0.53^b^ ± 0.01	0.76^b^ ± 0.06	− 0.53^b^ ± 0.01	1.15^a^ ± 0.09
h^º^	206.54^a^ ± 0.73	135.01^b^ ± 0.40	205.63^a^ ± 0.70	122.64^c^ ± 0.20
C*	1.20^a,b^ ± 0.01	1.07^b^ ± 0.01	1.26^a,b^ ± 0.01	1.36^a^ ± 0.07
**ΔE**				
	3.20		2.29	

Different letters on the same line differ statistically at the 95% confidence interval (*p* < 0.05).

In Table 4 (GAE: gallic acid equivalent, TE: trolox equivalents (Trolox-Equivalent Antioxidant Capacity), ABTS: 2,2′-azino-bi(3-ethylbenzothiazoline-6-sulfonic acid), DPPH: 2,2-Diphenyl-1-picrylhydrazyl, FRAP: ferric reducing antioxidant power, nd: not detected, < DL: values below detection limit, L*, luminosity; a*[(−) green to (+) red] and b* [(−) blue to + yellow] chromaticity coordinates, ho, Hue angle and C*, Chromaticity. ΔE: Total color difference. Different letters on the same line differ statistically at the 95% confidence interval (*p* < 0.05).

The pH ranged from 3.45 to 3.65, which is similar to that reported by Roda et al.^9^ and Chalchisa and Dereje^32^ in pineapple peel vinegar; pH values of 3.0 and 3.5, respectively.

Peel vinegar had lower acidity (4.5%) than the pulp vinegar (5.5%), as judged by the lower content of acetic acid present in these vinegars (Fig. 2a,b). Roda et al.^9^ and Raji et al.^47^ observed similar acidity values ​​(5.0% and 4.77%, respectively) in the vinegars of pineapple peel.

Brazilian legislation establishes that the minimum volatile acidity of commercially-produced vinegars must be equivalent to 4.0 g of acetic acid in 100 mL of the product. Acidity in acetic acid is a parameter of importance as it reflects the quality of vinegar, since it influences the flavor and acceptability of the product. Vinegar with acidity higher than 5.5% is commonly not acceptable by consumers. On the other hand, vinegar with low acidity produced by the traditional fermentation method is susceptible to contamination by the nematode, *Anguillula aceti* (vinegar eels)^33^.

The content of residual ethanol present in pineapple pulp vinegar (0.97%, v/v), pulp vinegar enriched with red-Jambo extract (0.97%, v/v), peel vinegar (0.61%, v/v), and peel vinegar enriched with this extract (0.97%, v/v) are in accordance with Brazilian legislation, which establishes maximum values ​​of 1% ethanol (v/v). The values ​​found were similar to those reported by Roda et al.^9^ in pineapple peel vinegar (0.50% v/v). It should be noted that small amounts of residual ethanol are necessary since acetic bacteria can promote the degradation of acetic acid in the absence of ethanol^48^.

Fixed mineral residue (ash) values ​​in fruit vinegar are also established by Brazilian legislation, which must be at least 1 g/L, and in this sense, all commercial vinegar produced presents adequate values. The total dry extract parameter refers to the content of minerals and organic matter that persist after evaporating water and volatile substances from the vinegar^33^. The values ​​found in pulp vinegar are close to those described in the literature for fruit vinegars. The values ​​of the total dry extract were lower in the vinegar samples obtained from the peels than compared to the pulp vinegar samples. This was probably due to the dilution of the peels in obtaining the must for vinification and wine production. Different contents of total dry extract in fruit peel vinegar have been reported in the literature. Prisacaru et al.^49^ reported values ​​between 2.11 g/100 mL and 26.43 g/100 mL, while values between 6.90 g/L and 10.59 g/L were mentioned by^50^. The Brazilian legislation determines a minimum amount of 6 g/L of reduced dry extract in fruit vinegars, the values ​​found in the present work within the current legislation limit.

Regarding density, the vinegar of pulp (1.024 g/mL), peels (1.026 g/mL), pulp with plant extract (1.066 g/mL), and peels with plant extract (1.011 g/mL) are in agreement with what was reported by Raji et al.^47^ in pineapple peel vinegar (1.08 g/mL). High amounts of phenolic compounds (Pulp Vinegar: 364.45 mg GAE/L, Peel Vinegar: 222.94 mg GAE/L, Pulp + Extract- Vinegar: 443.60 mg GAE/L and Peel + Extract- Vinegar: 337.63 mg GAE/L) were observed in all of the pineapple vinegar samples. It should be noted that pineapple peel vinegar, however, had lower total phenolic contents than those found in the pulp vinegar. In fact, the pulp wine used in acetic fermentation already had a higher content of phenolic substances from the pineapple fruit itself. Another interesting aspect observed was that the wine acetification process increased the phenolic content. This phenomenon occurred because acetic fermentation was conducted by the traditional vinegar system produced in wooden vats. Phenolic compounds migrate from the wooden walls of the acetification barrel and into the vinegar. The compounds supplied by the wood will depend on the type of wood and the roasting of the barrel, the relationship between the contact surface and the volume of liquid, and the contact time^51^.

The addition of red-Jambo extract in pineapple pulp and peel vinegars promoted the enrichment of the total phenolic content. Among the phenolic substances identified in the samples, the highest epicatechin concentrations were found in the pulp vinegar plus the extract (34.36 mg/L). Caffeic acid (14.89 mg/L) was peel vinegar's most prominent phenolic compound. Gallic (862.61 µg/mL) and caffeic (218.91 µg/mL) acids were reported by Mohamad et al.^52^ as the major phenolic compounds in pineapple pulp vinegar.

In addition to having high contents of total phenolics, the vinegar produced also had an appreciable ability to scavenge DPPH and ABTS radicals and ferric reducing antioxidant power (FRAP). Regarding the capture capacity of the ABTS radical, values ​​of 410.5 µmol TE/100 mL (Pulp Vinegar), 266.6 µmol TE/100 mL (Peel Vinegar), 547.1 µmol TE/100 mL (Pulp + Extract Vinegar) 337.5 µmol TE /100 mL (Peel + Extract Vinegar) were obtained. The ABTS radical scavenging potential of the pulp vinegar was higher for the peel vinegar. Similarly, pulp vinegar showed greater ferric ion reducing potential (Pulp Vinegar: 402.8 µmol TE/100 mL and Pulp + Extract Vinegar: 675.8 µmol TE/100 mL) compared to peel vinegars (Peel Vinegar: 277.8 µmol TE/100 mL and Peel + Extract Vinegar: 542.3 µmol TE/100 mL).On the other hand, peel vinegar was more efficient in capturing the DPPH radical (peel vinegar: 227.8 µmol TE/100 mL and peel + extract vinegar: 277.5 µmol TE/100 mL) than pulp vinegar (Pulp Vinegar: 216.4 µmol TE/100 mL and Pulp + Extract Vinegar: 249.8 µmol TE/100 mL). The chemical nature of the bioactive compounds present in the vinegars, including chemical structure, polarity and hydrophobicity, strongly influence their free-radical scavenging capacity or reducing antioxidant potential. In this sense, more than one method is commonly used to evaluate the antioxidant potential of the same sample since the antioxidant evaluation methods are correlated with the mechanisms of antioxidant action^53^. Several studies of antioxidant activity in vinegar samples have been described in the scientific literature. However, there is some difficulty in comparing results due to the diversity of methods and expression of results. Fonseca et al.^17^ reported similar values ​​for the scavenging of ABTS (368.39–402.15 μmol TE/100 mL) and DPPH (186.73–211.39 μmol TE/100 mL) radicals in blueberry and honey vinegar. Regarding the FRAP potential, these authors found much higher values ​​(1881.45–1884.5 μmol FeSO_4_/100 mL) in relation to the vinegar obtained in the present study.

The same organic acids present in the pineapple pulp and peel wines were found in the vinegar samples. Like what was observed in pulp wines, citric (pulp vinegar: 7.41 g/L and pulp + extract vinegar: 7.46 g/L) and oxalic (pulp vinegar: 9.04 g/L and pulp + extract vinegar: 9.09 g/L) acids were the predominant organic acids in pulp vinegar. Oxalic acid (peel vinegar: 4.75 g/L and peel + extract vinegar: 4.78) and citric acid (peel vinegar: 1.46 g/L and peel + extract vinegar: 1.48 g/L) predominated in pineapple peel vinegar. The enrichment of vinegar with red-Jambo leaf extract did not promote statistically significant changes in the composition of organic acids.

Pineapple pulp vinegar showed luminosity (L^*^: 45.39) close to the spectrum observed in wine (L^*^: 42.73). On the other hand, the peel vinegar (with and without the addition of red-Jambo leaf extract) showed slightly lower luminosity values ​​(peel vinegar: 42.29 and peel + extract vinegar: 43.12) than those found in the peel wine samples (L^*^: 52.75). The reduction in luminosity values ​​indicates that the acetic fermentation process carried out in wooden barrels contributes to a certain decrease in the perception of brightness and clarity of the peel vinegar samples. This phenomenon may be associated with the probable extraction of compounds from the wood of the acetification barrel, associated with the chemical composition of these vinegars, which present differences in the concentrations of phenolic compounds compared to pulp vinegar. The color properties of different types of vinegar can change depending upon the color of the raw material and the technology used in the production^54^***.***

Regarding the a^*^ coordinate (red index), the acetic fermentation contributed to a green tendency, which was more pronounced in the peel vinegar samples. Similarly, the acetification of wines also reduced the values ​​of the b^*^ coordinate (yellow index: tendency from yellow to blue), especially in the vinegar of peels. The acetification of the wines led to an intensification of the hº coordinate (hue angle), increasing the tendency to green (from yellow to green) in the peel vinegar samples (peel vinegar:135.01 and peel + extract vinegar: 122.64). A more pronounced increase in the values ​​of the hº coordinate was observed in the pulp vinegar samples in relation to the pulp wines, with a tendency to intensify the blue hue (peel vinegar: 206.54, peel + extract vinegar: 205.63). The evaluation of the chromaticity index (C^*^) of the samples indicates that the acetification led to the reduction of the saturation index of the vinegar samples concerning the wines, this phenomenon being more pronounced in the vinegar of pineapple peels. The enrichment of pulp vinegar with red-Jambo leaf extract did not promote statistically significant changes in the color parameters of the pulp vinegar samples (L^*^ a^*^ b^*^, hº, and C^*^). On the other hand, adding the leaf extract to the peel vinegar led to changes in the instrumentally-detectable color parameters (L^*^ b^*^, h^º^, and C^*^) (peel vinegar versus peel + extract vinegar).

It is essential to highlight that the evaluation of the total color difference (ΔE) indicates that adding the red-Jambo leaf extract to the samples did not promote visually-detectable changes when comparing samples with or without added extract. Some reports in the scientific literature and based on the study described by Stokes et al.^55^ mention that color differences less than 2.15 are not perceptible to the human eye^44^. The results of ΔE found in the present work are close to this threshold, especially in relation to vinegar obtained from pineapple peels (Table 4).

### Antimicrobial potential

Vinegar has been recognized as an antimicrobial substance for a long time, and several studies have shown vinegars of different origin can act as antimicrobial agents against different pathogens^54^*.* Table 5 shows the results of the susceptibility of different bacterial strains (gram-negative and gram-positive) and yeasts to the pulp and peel vinegar produced in the present work. Diffusion disk tests and evaluation of the minimum inhibitory concentration (MIC) showed that all vinegars, with or without added red-Jumbo leaf extract in the present study, presented inhibition against the microorganisms studied.

**Table 5:** Antimicrobial and antifungal potential of pineapple vinegar samples.

Microorganism	Vinegar samples					
	Pulp	Peel	Pulp + extract	Peel + extract	Alcohol^&^	AS
*Staphylococcus aureus* ATCC 25923						
ID (mm)	14.67^a, c^ ± 0.80	7.33^a^ ± 1,80	23.03^a, c^ ± 2.70	13.01^a, c^ ± 1.30	17.60^c^ ± 1.50	42.0^b^ ± 0.5
MIC (µL/mL)	16.0	16.0	16.0	16.0	*	*
MBC (µL/mL)	50.5	50.5	50.5	16.0	*	*
*Escherichia coli* ATCC 25922						
ID (mm)	14.02^b^ ± 0.70	8.33^b^ ± 0.90	17.67^b^ ± 3.10	15.67^b^ ± 2.90	13.7 ^b^ ± 1.8	50.0^a^ ± 0.5
MIC (µL/mL)	5.0	16.0	5.0	16.0	*	*
MBC (µL/mL)	#	#	#	#	*	*
*Salmonella enterica* typhimurium ATCC 19,659						
ID (mm)	13.03^a, b^ ± 2.00	5.66^b^ ± 0.80	14.02^a, c^ ± 1.30	12.33^b, c^ ± 1.50	12.0^b, c^ ± 1.1	44.0^a^ ± 0.8
MIC (µL/mL)	16.0	16.0	16.0	16.0	*	*
MBC (µL/mL)	151.5	50.5	151.5	50.5	*	*
*Bacilus subtilis* ATCC 0028						
ID (mm)	12.66^b^ ± 0.90	9.01^b^ ± 1.10	18.7^a, b^ ± 3.7	12.5^b^ ± 1.3	10.0^b^ ± 0.6	36.0^a^ ± 0.9
MIC (µL/mL)	5.0	16.0	5.0	16.0	*	*
MBC (µL/mL)	16.0	50.5	16.0	50.5	*	*
*Candida albicans* ATCC 118,804						
ID (mm)	20.0^b^ ± 4.4	16.0^b^ ± 2.8	20.0^b^ ± 2.2	12.0^b^ ± 1.7	12.0^b^ ± 1.5	50.0^a^ ± 1.0
MIC (µL/mL)	16.0	16.0	16.0	16.0	*	*
MFC (µL/mL)	50.5	151.5	50.5	151.5	*	*
*Candida tropicalis* ATCC13803						
ID (mm)	20.0^b^ ± 2.2	20.0^b^ ± 1.5	22.0^b^ ± 1.1	22.0^b^ ± 2.0	22.0^b^ ± 1.7	50.0^a^ ± 0.9
MIC (µL/mL)	16.0	16.0	16.0	16.0	*	*
MFC (µL/mL)	50.5	50.5	50.5	50.5	*	*

AS, standard antimicrobial, tetracycline for bacteria and fluconazole for fungi); ID, diameter of the inhibition zones (inhibition halo); MIC, minimum inhibitory concentration; MBC, minimum bactericidal concentration; MFC, minimum fungicide concentration.

*, not evaluated; ^*#*^, no inhibition; ^*&*^, commercial alcohol vinegar.

In Table 5 (AS: standard antimicrobial, Tetracycline for bacteria and Fluconazole for fungi), ID: Diameter of the inhibition zones (inhibition halo), MIC: minimum inhibitory concentration, MBC: minimum bactericidal concentration, MFC: Minimum fungicide concentration, *: no evaluated, ^*#*^: no inhibition, ^***&***^: commercial alcohol vinegar).

Pineapple pulp vinegar promoted greater inhibition halos against both bacteria and yeasts when compared to peel vinegar. Diffusion disk tests also showed that the enrichment of vinegar with red-Jambo leaf extract potentiated the antimicrobial activity. Another aspect observed is that the pulp and peel vinegars promoted inhibition diameters similar to those observed using commercial alcohol vinegar (4.0 g acetic acid/100 mL).

The minimum concentrations necessary to inhibit the microorganisms studied ranged from 5 µL/mL to 16 µL/mL. This range was wider regarding the bactericidal concentration, ranging from 15.5 µL/mL to 151.5 µL/mL. Similarly, the concentrations required for yeast inhibition ranged from 16 µL/mL to 151.5 µL/mL. These values ​​indicate that the sensitivity of the microorganisms studied against the pineapple vinegar samples was relatively variable. In agreement with the results obtained, Ozturk et al.^56^ reported a high variability in a study with twenty samples of traditional vinegar produced in Turkey (homemade vinegar) in relation to the sensitivity of the bacteria studied.

Bacillus subtilis (G^+^) was the microorganism most sensitive to pure pulp vinegar and enriched with the red-Jambo leaf extract, with inhibition at a 5.0 µL/mL concentration and cell death at a concentration of 16.0 µL/mL. Escherichia coli (G^−^) was the most resistant strain, being inhibited at a concentration of 5.0 µL/mL in pulp vinegar (with or without the added red-Jambo leaf extract) and 16.0 µL/mL in peel vinegar (with or without leaf extract), but showed resistance to the biocidal activity of the vinegar samples. Similarly, Ousaaid et al.^57^ reported E. coli as the microbial strain most resistant to antimicrobial activity (MIC: 3.125 μL/mL; MBC: 6.25 μL/mL) in apple cider vinegar.

In evaluating the antimicrobial capacity against the yeasts, we observed that the pineapple pulp vinegar showed more significant antimicrobial potential against the Candida albicans strain than the peel vinegar. Pulp vinegar was able to inhibit this yeast at a 16.0 µL/mL concentration and promote its death at a 50.0 µL/mL concentration. However, there was no observable antimicrobial potentiation of the red-Jumbo leaf extract against the two Candida tropicalis yeast strains evaluated, thus maintaining its minimum inhibitory concentration and fungicidal concentration.

The enrichment of peel vinegar with red-Jambo leaf extract potentiated the antimicrobial activity against the Gram-positive bacterium S. aureus, with MBC values ranging from 50.5 μL/mL (peel vinegar) to 16 μL/mL (peel + extract vinegar). On the other hand, adding the red-Jumbo leaf extract to the vinegar did not potentiate the antimicrobial activity against the other microorganisms. The antimicrobial potential of vinegar is associated with the presence of organic acids, which have antimicrobial activity, especially acetic acid, which can cross the bacterial membrane and promote a reduction in intracellular pH, consequently causing the death of the microorganism^57^. Weak organic acids cross the cell membrane in the undissociated form and dissociate according to intracellular pH, releasing a proton into the cytoplasm^56^.

## Conclusion

The production of wines and vinegars from pineapple pulp and peels can be a strategic tool for using the entire fruit within a circular economy context. Red-Jambo (*Syzygium malaccense*) leaf extract studied was rich in phenolic compounds and flavonoids and showed high antioxidant potential in vitro. The yeast strain *Saccharomyces cerevisiae* r. f. bayanus showed good efficiency in the alcoholic fermentation of pineapple pulp- and peel- based musts. The pulp and peel wines presented appreciable contents of total phenolic compounds, phenolic acids, flavonoids, and organic acids, as well as high antioxidant potential. Higher amounts of phenolic compounds and higher antioxidant capacity were found in pineapple pulp and peel vinegars than in wines indicating that the acetification process contributed to the potentiation of bioactive properties. Adding red-Jambo leaf extract to vinegar contributed to the enrichment of total phenolic compounds and antioxidant activity, and potentiated the antimicrobial activity observed. The pineapple-derived vinegars showed antimicrobial activity with biocidal action against the bacteria *Staphylococcus aureus*, *Bacillus subtilis*, *Salmonella enterica* Typhimurium, and the yeasts *Candida tropicalis* and *Candida albicans*. The vinegars produced can be considered specialy vinegars, and their production can contribute to the use of vegetable biomass that is commonly discarded or underused during processing, adding value to the production chain. As a future perspective that could attract consumers and expand the vinegar market, our study highlights using red-Jambo leaf extracts rich in bioactive compounds, including compounds associated with digestive properties.

## Compliance

The procedures for collecting and obtaining the studied plant extract followed the relevant institutional, national, and international guidelines and legislation. The voucher specimen was prepared and identified by botanist Dr. Giovana Faneco Pereira, the copy remaining deposited in the Herbarium of the Universidade Tecnológica Federal do Paraná, Campus Pato Branco, under the collection number HPB 1173.

## Data Availability

The datasets generated during and/or analyzed during the current study are available from the corresponding author on reasonable request.

## References

[1] Cunha, M. A. A., Sene, L., Bertan, F. A. B. & Prado Martin, J. G. Microbiologia da fermentação de vinagre. In *Microbiologia de alimentos fermentados* (eds. Prado Martin, J. G. & de Dea Lindner, J.) 704 (Edgard Blücher, 2022).

[2] Singh AK (2020). Overview of vinegar production. PalArch’s J. Archaeol. Egypt Egyptol..

[3] Román-Camacho JJ (2020). Metaproteomics of microbiota involved in submerged culture production of alcohol wine vinegar: A first approach. Int. J. Food Microbiol..

[4] Taweecheep P (2019). In vitro thermal and ethanol adaptations to improve vinegar fermentation at high temperature of *Komagataeibacter oboediens* MSKU 3. Appl. Biochem. Biotechnol..

[5] Nie Z (2017). Unraveling the correlation between microbiota succession and metabolite changes in traditional Shanxi aged vinegar. Sci. Rep..

[6] Li S, Li P, Feng F, Luo L-X (2015). Microbial diversity and their roles in the vinegar fermentation process. Appl. Microbiol. Biotechnol..

[7] Zhang Q (2020). Monitoring microbial succession and metabolic activity during manual and mechanical solid-state fermentation of Chinese cereal vinegar. LWT.

[8] Zhang X-L (2020). Knowledge domain and emerging trends in vinegar research: A bibliometric review of the literature from WoSCC. Foods.

[9] Roda A (2017). Metabolite profiling and volatiles of pineapple wine and vinegar obtained from pineapple waste. Food Chem..

[10] Lima PCC, Souza BS, Oliveira ATSDC (2017). Aproveitamento agroindustrial de resíduos provenientes do abacaxi pérola minimamente processado. Holos.

[11] Sarangi PK (2022). Sustainable utilization of pineapple wastes for production of bioenergy, biochemicals and value-added products: A review. Bioresour. Technol..

[12] Kumar P (2021). Pineapple peel extract incorporated poly(vinyl alcohol)-corn starch film for active food packaging: Preparation, characterization and antioxidant activity. Int. J. Biol. Macromol..

[13] Xu Y, Liu C, Qu Y, Ding Y, Zhang J (2022). Modified pineapple peel extract coupled with electrokinetic techniques for remediation of chromium-contaminated soil. Process Saf. Environ. Prot..

[14] Ajayi AM, Coker AI, Oyebanjo OT, Adebanjo IM, Ademowo OG (2022). *Ananas comosus* (L.) Merrill (pineapple) fruit peel extract demonstrates antimalarial, anti-nociceptive and anti-inflammatory activities in experimental models. J. Ethnopharmacol..

[15] Arumugam B, Manaharan T, Heng CK, Kuppusamy UR, Palanisamy UD (2014). Antioxidant and antiglycemic potentials of a standardized extract of *Syzygium malaccense*. LWT Food Sci. Technol..

[16] Fernandes, F. A. N. & Rodrigues, S. Jambo-*Syzygium malaccense*. In *Exotic Fruits* (eds. Rodrigues, S., Oliveira Silva, E. & Sousa de Brito, E.) 245–249 (Elsevier, 2018). 10.1016/B978-0-12-803138-4.00031-9.

[17] daFonseca MS (2018). Blueberry and honey vinegar: successive batch production, antioxidant potential and antimicrobial ability. Braz. J. Food Technol..

[18] Savi A (2020). Bioactive compounds from *Syzygium malaccense* leaves: Optimization of the extraction process, biological and chemical characterization. Acta Sci. Technol..

[19] Miller GL (1959). Use of dinitrosalicylic acid reagent for determination of reducing sugar. Anal. Chem..

[20] Singleton VL, Orthofer R, Lamuela-Raventós RM (1998). Analysis of total phenols and other oxidation substrates and antioxidants by means of folin-ciocalteu reagent. Methods Enzymol..

[21] Budak HN, Guzel-Seydim ZB (2010). Antioxidant activity and phenolic content of wine vinegars produced by two different techniques. J. Sci. Food Agric..

[22] Brand-Williams W, Cuvelier ME, Berset C (1995). Use of a free radical method to evaluate antioxidant activity. LWT Food Sci. Technol..

[23] DoRufino MSM (2010). Bioactive compounds and antioxidant capacities of 18 non-traditional tropical fruits from Brazil. Food Chem..

[24] Instituto Adolfo Lutz. 1^a^ Edição Digital. Métodos físicos-quimicos para análise de Alimentos (Instituto Adolfo Lutz, 2008).

[25] Reanpang P, Pun-uam T, Jakmunee J, Khonyoung S (2021). An environmentally friendly flow injection-gas diffusion system using roselle (*Hibiscus sabdariffa* L.) extract as natural reagent for the photometric determination of sulfite in wines. J. Anal. Methods Chem..

[26] Batista ÂG (2017). Red-jambo (*Syzygium malaccense*): Bioactive compounds in fruits and leaves. LWT Food Sci. Technol..

[27] Savitha RC, Padmavathy S, Sundhararajan A (2011). Invitro antioxidant activities on leaf extracts of *Syzygium malaccense* (L.) Merr and Perry. Anc. Sci. Life.

[28] Campos DA, Ribeiro TB, Teixeira JA, Pastrana L, Pintado MM (2020). Integral valorization of pineapple (*Ananas comosus *L.) by-products through a green chemistry approach towards added value ingredients. Foods.

[29] Lubaina AS, Renjith PR, Kumar P (2019). Antibacterial potential of different extracts of pineapple peel against gram-positive and gram-negative bacterial strains. Asian J. Pharm. Pharmacol..

[30] Mulyani S, Ariani SRD, Utomo SB, Antrakusuma B (2021). Phytochemical screening of honey pineapple peel extract and its application as an antibacterial additive in dish soap formulation. J. Kim. Dan Pendidik. Kim..

[31] Alvarenga LM (2015). Analysis of alcoholic fermentation of pulp and residues from pineapple processing. CYTA J. Food.

[32] Chalchisa T, Dereje B (2021). From waste to food : Utilization of pineapple peels for vinegar production. MOJ Food Process. Technol..

[33] Cunha MAA, Lima KPDE, Santos VAQ, Heinz OL, Schmidt CAP (2016). Blackberry vinegar produced by successive acetification cycles : Production, characterization and bioactivity parameters. Braz. Arch. Biol. Technol..

[34] Qi N, Ma L, Li L, Gong X, Ye J (2017). Production and quality evaluation of pineapple fruit wine. IOP Conf. Ser. Earth Environ. Sci..

[35] Akanni AL (2015). Valorization of pineapple produced in Benin: Production and evaluation of wine quality parameters. Int. J. Adv. Res. J..

[36] Queiroz, G. A. de, Rabelo, A. G. S. & Santos, S. K. M. Characterization and optimization of production process of alcoholic fermentation of pineapple. *Rev Eletrônica em Gestão Educ e Tecnol. Ambient.* **23**, 30 (2019).

[37] Agência Nacional de Vigilância Sanitária. *Resolução da Diretoria Colegiada - RDC N° 123, de 4 de Novembro de 2016*. 56–57 (2016).

[38] Pino JA, Queris O (2010). Analysis of volatile compounds of pineapple wine using solid-phase microextraction techniques. Food Chem..

[39] Zhang L (2020). Physicochemical characterization of pineapple peel wine. IOP Conf. Ser. Earth Environ. Sci..

[40] Li T (2014). Major polyphenolics in pineapple peels and their antioxidant interactions. Int. J. Food Prop..

[41] Lu XH, Sun DQ, Wu QS, Liu SH, Sun GM (2014). Physico-chemical properties, antioxidant activity and mineral contents of pineapple genotypes grown in China. Molecules.

[42] Cordenunsi B (2010). Carbohydrate composition of ripe pineapple (cv. perola) and the glycemic response in humans. Ciência e Tecnol. Aliment..

[43] Batista-Silva W (2018). Modifications in organic acid profiles during fruit development and ripening: Correlation or causation?. Front. Plant Sci..

[44] Nissola C (2021). Hydrogel containing (1→6)-β-D-glucan (lasiodiplodan) effectively promotes dermal wound healing. Int. J. Biol. Macromol..

[45] Tanamool V, Chantarangsee M, Soemphol W (2020). Simultaneous vinegar fermentation from a pineapple by-product using the co-inoculation of yeast and thermotolerant acetic acid bacteria and their physiochemical properties. 3 Biotech..

[46] Iurckevicz G (2021). Bioactive compounds in the leaves of *Baccharis dracunculifolia*: Extraction process and characterization. Acta Sci. Technol..

[47] Raji YO, Mohammed J, Misau I, Baba YD (2012). Production of vinegar From pineapple peel. Int. J. Adv. Sci. Res. Technol..

[48] Gomes RJ, De Borges MF, De Rosa MF, Castro-Gómez RJH, Spinosa WA (2018). Acetic acid bacteria in the food industry: Systematics, characteristics and applications. Food Technol. Biotechnol..

[49] Prisacaru AE, Ghinea C, Apostol LC, Ropciuc S, Ursachi VF (2021). Physicochemical characteristics of vinegar from banana peels and commercial vinegars before and after in vitro digestion. Processes.

[50] Viroli SLM, Viroli SG, Carvalho NP, DaBernardi DPS, Coelho RG (2021). Production and characterization of acetic fermentation with different fruit peels. Res. Soc. Dev..

[51] Mas A, Torija MJ, DelGarcía-Parrilla MC, Troncoso AM (2014). Acetic acid bacteria and the production and quality of wine vinegar. Sci. World J..

[52] Mohamad NE (2015). Antioxidant effects of pineapple vinegar in reversing of paracetamol-induced liver damage in mice. Chin. Med. (UK).

[53] Ivanova A, Gerasimova E, Gazizullina E (2020). Study of antioxidant properties of agents from the perspective of their action mechanisms. Molecules.

[54] Kılıç G, Şengün İY (2021). Fig vinegar as an antioxidant and antimicrobial agent. Turk. J. Agric. Food Sci. Technol..

[55] Stokes M, Fairchild MD, Berns RS (1992). Precision requirements for digital color reproduction. ACM Trans. Graph..

[56] Ozturk I (2015). Antioxidant, antimicrobial, mineral, volatile, physicochemical and microbiological characteristics of traditional home-made Turkish vinegars. Lwt.

[57] Ousaaid D, Imtara H, Laaroussi H, Lyoussi B, Elarabi I (2021). An investigation of Moroccan vinegars: Their physicochemical properties and antioxidant and antibacterial activities. J. Food Qual..

